# Gelatin Nanoparticles for Targeted Dual Drug Release out of Alginate-di-Aldehyde-Gelatin Gels

**DOI:** 10.3390/gels8060365

**Published:** 2022-06-08

**Authors:** Sophie Schrade, Lucas Ritschl, Regine Süss, Pia Schilling, Michael Seidenstuecker

**Affiliations:** 1 G.E.R.N. Center of Tissue Replacement, Regeneration & Neogenesis, Department of Orthopedics and Trauma Surgery, Medical Center—Albert-Ludwigs-University of Freiburg, Faculty of Medicine, Albert-Ludwigs-University of Freiburg, Hugstetter Straße 55, 79106 Freiburg, Germany; sophie.schrade@uniklinik-freiburg.de (S.S.); lucas.ritschl@uniklinik-freiburg.de (L.R.); pia.schilling@uniklinik-freiburg.de (P.S.); 2 Institute of Pharmaceutical Sciences, Albert-Ludwigs-University of Freiburg, Sonnenstr. 5, 79104 Freiburg, Germany; regine.suess@pharmazie.uni-freiburg.de

**Keywords:** gelatin nanoparticle, controlled drug release, antibiotics, growth factor, ADA-gelatin

## Abstract

The aim of the present work was to develop a dual staged drug release of an antibiotic (clindamycin) and a growth factor: bone morphogenetic protein-2 (BMP-2) from a biodegradable system consisting of hydrogel and gelatin nanoparticles (GNP). Two-step de-solvation allowed us to prepare GNPs (~100 nm) as drug carriers. Fluorescein isothiocyanate (FITC)-conjugated protein A was used as a model substance for BMP-2. A 28-day release experiment was performed to determine the release kinetics from GNP for both FITC-protein A and BMP-2, and for clindamycin (CLI) from the hydrogel. The size, structure, and overall morphology of GNP samples (empty, loaded with FITC-protein A and BMP-2) were examined using an environmental scanning electron microscope (ESEM). Cell culture assays (Live/dead; cell proliferation; cytotoxicity) were performed with MG-63 cells and BMP-2-loaded GNPs. Drug release experiments using clindamycin-loaded alginate-di-aldehyde (ADA) gelatin gels containing the drug-loaded GNPs were performed for 28 days. The resulting GNPs showed an empty size of 117 ± 29 nm, 176 ± 15 nm and 216 ± 36 nm when containing 2% FITC-protein A and 1% BMP-2, respectively. No negative effects of BMP-2-loaded GNPs on MG-63 cells were observed in live/dead staining. In the proliferation assay, an increase in cell proliferation was observed for both GNPs (GNP + BMP-2 and controls). The cytotoxicity assay continuously showed very low cytotoxicity for GNPs (empty; loaded). Clindamycin release showed a concentration of 25-fold higher than the minimum inhibitory concentration (MIC) against Staphylococcus aureus throughout the 28 day period. BMP-2 showed a reduced burst release and a steady release (~2 µg/mL) over a 28 day period.

## 1. Introduction

Bone infections, such as osteomyelitis, often present a challenge in trauma surgery and orthopaedic settings. The therapy is often lengthy and requires a high degree of diagnostic and therapeutic experience on the part of the treating physician. Osteomyelitis is an infection of the bone and surrounding soft tissues. As a rule, the infection is bacterial in nature, but fungi and viruses can also cause osteomyelitis [1,2]. Osteitis can be divided into acute post-traumatic, chronic post-traumatic and acute haematogenic osteomyelitis, the latter being of endogenous origin and rare in adults. Acute haematogenic osteomyelitis is more common in children and adolescents, with one third of cases occurring in children under 2 years of age. The germs usually enter the bloodstream through infections in the nasopharynx and can thus attack the bone from the inner medullary cavity [3]. In an exogenous infection the germs penetrate via an injury to the exterior of the bone. Post-traumatic or post-operative infections occur mainly after open fractures and surgical interventions such as the insertion of endoprostheses into the body. Open fractures present an increased risk of developing a bone infection. The risk of post-traumatic infection can rise to 25% depending on the type of fracture, the amount of bone loss, bacterial contamination, the degree of soft tissue injury and other injuries, such as damage to local vasculature [4,5]. Periprosthetic infection is a common complication after implantation of an endoprosthesis [6]. Due to the increasing age of the population and the growing desire not to lose mobility even in old age, the number of implantations of such total endoprostheses (TEP) is rising sharply [7] and thus also the number of postoperative infections. 2019 alone (as this, COVID-19 related, is the most reliable data of the last years [8]), 243,477 TEPs were implanted in the hip joint and 193,759 in the knee joint in Germany [9]. In the USA, 498,000 TEPs were implanted in the hip joint and 1,065,000 total knee replacement surgeries were performed during the same period [10]. Further implantations of such TEPs are performed on the shoulder, elbow and ankle joint. It is expected that infection will manifest itself in 17% of the procedures of such TEPs. As a rule, the bacteria usually reach the surface of the implants during the surgical procedure, although less frequently they may also stem from endogenous sources [11]. The bacterium *Staphylococcus epidermidis*, which is part of the normal skin flora, often colonizes the surface of medical devices such as catheters and implants with a biofilm [12]. The surface of such implants offers the bacteria the opportunity to form a biofilm by changing from a planktonic to a sessile form. In the sessile form the bacteria do not grow at all or grow very slowly and are therefore not attacked by most antibiotics. The biofilm protects the bacteria from phagocytosis of the immune cells and represents a diffusion barrier for the antibiotics [13]. Periprosthetic osteomyelitis develops from a periprosthetic infection in 20% of cases. Depending on the risk factors present, about 10–30% of acute osteomyelitis develop into chronic forms [14]. Patients with chronic osteomyelitis often have a long history of suffering with frequent recurrences and sometimes a life-long illness [15]. Frequent hospitalisation and loss of employment severely restrict the quality of life of patients and the economic impact of such chronic infections is also severe. The most common pathogen causing osteomyelitis is *Staphylococcus aureus* (*S. aureus*) [16], a gram-positive, spherical bacterium which, like *Staphylococcus epidermidis*, is part of the skin and mucous membrane flora and can also form a biofilm. Besides *Escherichia coli* (*E. coli*), *S. aureus* causes the most common bacterial infections in humans [17]. In addition to the many resistances to common antibiotics, the bacterium brings along further “tools” that considerably increase its pathogenicity. Cell wall-associated proteins such as fibronectin binding proteins and collagen binding proteins help *S. aureus* adhere to cells, tissue and foreign bodies. A number of extracellular enzymes such as hyaluronidases, lipases and plasma coagulases degrade tissue and ensure its dissemination within the tissue. In addition to osteomyelitis, other enzymes and toxins can cause many other clinical pictures such as toxic shock syndromes, endocarditis and sepsis [18]. The current treatment strategy for chronic osteomyelitis includes surgical removal of the infected area (radical debridement) followed by systemic or local antibiotic therapy. In order to prevent resistance and to ensure a targeted therapy, it is important to detect the pathogen and to determine the sensitivity of the bacterium to the respective antibiotic by means of an antibiogram. The antibiotic used should be bone-compatible and well tolerated by the patient. However, systemic antibiotic therapy reaches its limits in case of a bone infection. On the one hand, the biofilm protects the bacteria from the penetration of antibiotics, on the other hand, the progressing course of the disease often makes antibiotic therapy ineffective. The progressive inflammation leads to necrotic tissue and thus to a reduced blood supply. So called sequesters are formed, which as avital bone tissue create ideal conditions for biofilm colonization [19]. All of these factors ensure that at the site of the infection no drug levels above the minimum inhibitory concentration (MIC) can be achieved by systemic antibiotic therapy.

Local antibiotic therapy, on the other hand, has the advantage that higher drug levels can be achieved at the site of infection, which if administered systemically would lead to toxic serum levels. Thus, with a suitable choice of active ingredient carrier, the active ingredient can be released over a longer period of time, for example over 4 weeks. Systemic side effects can thus be prevented and the concentration of the active substance at the site of infection is above the MIC for a sufficient amount of time to eliminate all bacteria, even in deeper areas of the bone. Local drug carriers can be divided into non-biodegradable and biodegradable systems. The current standard is the implantation of gentamycin-containing bone cement spheres. These polymethylmethacrylate (PMMA) spheres (e.g., Septopal^®^) are strung together in a chain and are not biodegradable. The PMMA chains are inserted into the bone cavities after debridement and usually remain there for 7–10 days [20]. After this period, the chain must be removed again and a second surgical procedure is performed, which in turn can lead to complications. A further disadvantage is the incomplete release of the gentamycin from the hydrophobic polymer matrix [21]. Biodegradable local drug carriers have the advantage that a second operation is not necessary. Biodegradable collagen fleeces loaded with gentamycin (Septocoll^®^) or collagen lyophilisates loaded with teicoplanin (Targobone^®^) can be used for local antibiotic therapy of bone infections [22]. However, a complete release of gentamycin-loaded collagens was determined after 4 days [23]. A constant release over several weeks however, is more desirable. Due to the fact that the current therapeutic options for the treatment of bone infections are still not free of weak points, further research on biodegradable, local drug carriers with adequate and controllable drug release will be required in the future.

The aim of the present work was to develop a dual, staggered drug release of both an antibiotic and a growth factor utilizing such a biodegradable system. The lincosamide clindamycin (CLI) was used as an antibiotic and should be released first to eliminate the pathogens causing the infection. Subsequently, the growth factor Bone Morphogenetic Protein 2 (BMP-2) should be released, which promotes the formation of new bone and cartilage. CLI was to be released uniformly over a period of 4 weeks, so it was placed in the hydrogel for delayed release. The ADA-GEL was then placed in a microporous ceramic (β-TCP, as described in earlier studies) that was intended to serve as a drug delivery device. The hydrogel used in the present study was alginate-dialdehyde (ADA) combined with gelatin through the formation of Schiff’s bases. The combination of ADA with gelatin has established itself as particularly suitable, as it shows good stability and adequate degradation behavior. The prolonged release of clindamycin can be achieved by the molecules of the antibiotic first diffusing through the gel to the edge of the ceramic and then gradually being released. The BMP-2 was intended to be released with a delay and was therefore enclosed in gelatin nanoparticles (GNPs), which in turn were incorporated into the gel containing CLI. The ceramic was loaded with the CLI-containing ADA-gelatin gel, which contained gelatin nanoparticles with BMP-2 enclosed, via a vacuum-induced flow. Initially, a method was developed to produce GNPs in uniform shape and size (having a diameter of approximately 100 nm) reproducibly by two-step de-solvation [14,22]. In a further step, these nanoparticles were loaded with proteins. The morphology of GNPs was studied by means of electron microscopy. Due to the high cost of BMP-2, protein A coupled with fluorescein isothiocyanate (FITC) was initially used as a model substance. The release kinetics from the GNPs were determined for both FITC-protein A and BMP-2 in a 28 day release experiment. The GNPs loaded with FITC-protein A and then in the further course of the experiments with BMP-2 were placed in ADA gelatin gels, which additionally contained CLI. The release experiments were performed directly from beads cast from this ADA gelatin gel containing CLI and FITC-protein A or BMP-2. The quantitative determination of clindamycin was done by high pressure liquid chromatography (HPLC) measurements and FITC-protein A levels were determined by fluorimeter and BMP-2 using an ELISA immunoassay. Biocompatibility was investigated by means of cell culture experiments. A live dead assay was performed on the MG-63 cells cultured with the GNP beads.

## 2. Results

### 2.1. Manufacturing Process of GNPs

**Figure 1:**
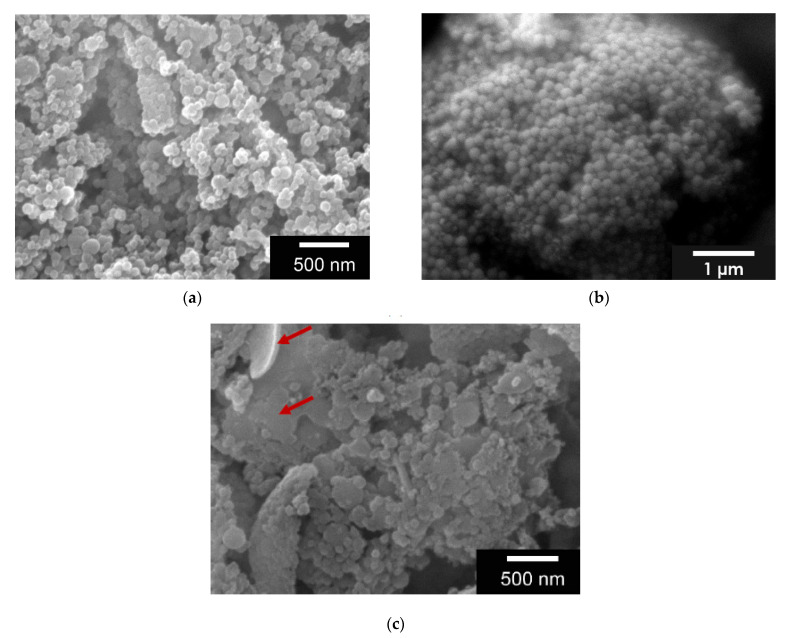
(**a**) ESEM Images of GNPs at a pH 2.0 which leads to GNPs smaller than 100 nm; (**b**) pH 2.5 with well-shaped GNPs having a size of 100 nm and (**c**) pH 3.0 with non-uniform size and shape of the GNPs, as well as structures not representing GNP (red arrow) Images taken with FEI Quanta 250 FEG at 12 kV acceleration voltage, all samples were sputter coated (JEOL, JFC-1200) with a 25 nm Gold layer.

The manufacturing method, in which the second de-solvation was performed at a pH of 2.5, provided the best results in terms of size and shape (117 ± 29 nm) and aggregation behavior. GNPs prepared at pH 3 could be resuspended very well but were more inconsistent in size and shape. Furthermore, the ESEM images also showed structures that were not of nanoparticulate form (see red arrows in Figure 1). GNPs produced at pH 2 were either too small (less than 100 nm) or the morphology was not spherical (see Figure 1).

The short precipitation time of 5 min after the first de-solvation was decisive for the success of the GNPs. The longer the wait after the first addition of acetone, the more likely more LMG precipitated into the HMG precipitate. This led to reticular and needle-like structures, but not to GNP production.

**Figure 2:**
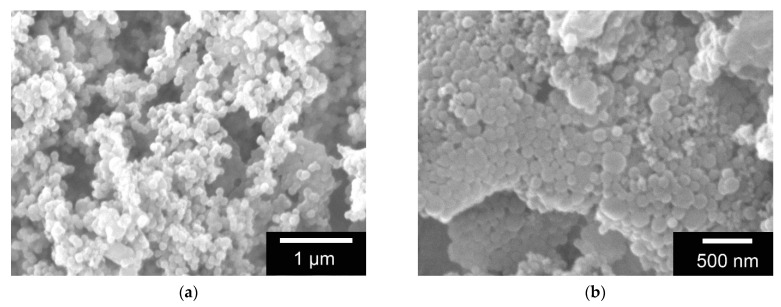
ESEM images of GNP with (**a**) 1% *w*/*v* FITC-protein A and (**b**) 2% *w*/*v* FITC-protein A. Images taken with FEI Quanta 250 FEG at 12 kV acceleration voltage, all samples were sputter coated (JEOL, JFC-1200) with a 25 nm Gold layer.

GNPs with FITC-protein A differed strongly in morphology and size. Hence, the GNPs of the mixture prepared with 1% FITC-protein A solution were very small (less than 50 nm), whereas those of the 2% mixture were on average about 176 ± 15 nm in size and were all uniformly spherical (see Figure 2).

**Figure 3:**
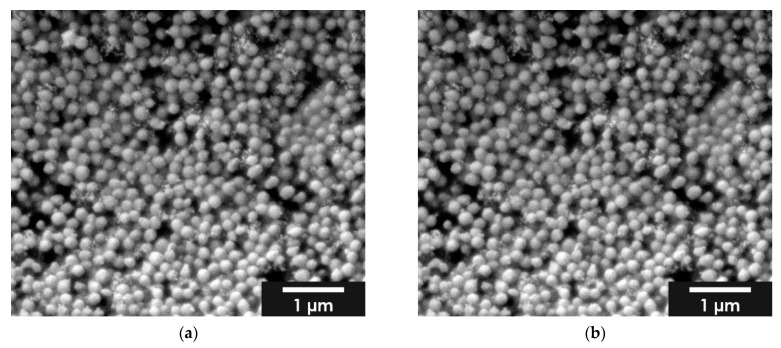
ESEM images of BMP-2 loaded GNP; (**a**) 1% *w*/*v* BMP-2; (**b**) 2% *w*/*v* BMP-2; images taken with FEI Quanta 250 FEG at 12 kV acceleration voltage, all samples were sputter coated (JEOL, JFC-1200) with a 25 nm Gold layer.

The GNPs loaded with BMP-2 were on average larger at 216 ± 36 nm than those with FITC-protein A (176 nm). The SEM images in Figure 3 show the formation of aggregates.

### 2.2. Inclusion Capacity

The inclusion capacity (IC) was determined as described in 2.3.2. A 6-point calibration curve was drawn (R^2^ = 0.9946) and the amount of FITC-protein A in the supernatant was determined. The inclusion capacity of the GNPs was 45.98%, with 250 μg FITC-protein A and 0.625 g GEL. The inclusion capacity of BMP-2 in the GNPs was 99.5% with 1 µg BMP-2 and 0.625 g GEL.

### 2.3. Biocompatibility

#### 2.3.1. Live Dead Staining

**Figure 4:**
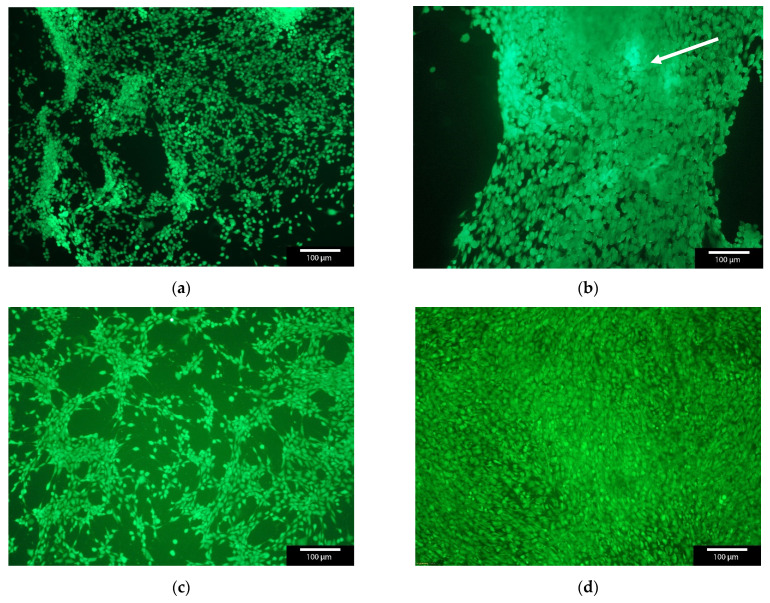
Live/Dead staining of MG-63 cells with GNPs versus MG-63 as control. The agglomerates of GNPs glowed slightly brighter in fluorescence microscopy (see white arrow); with GNPs after (**a**) 3 days; (**b**) 7 days; (**c**) control after 3 days; and (**d**) control after 7 days; green—living cells, red—dead cells. Images taken with Olympus BX-51 Fluorescence microscope, 5× magnification.

**Figure 5:**
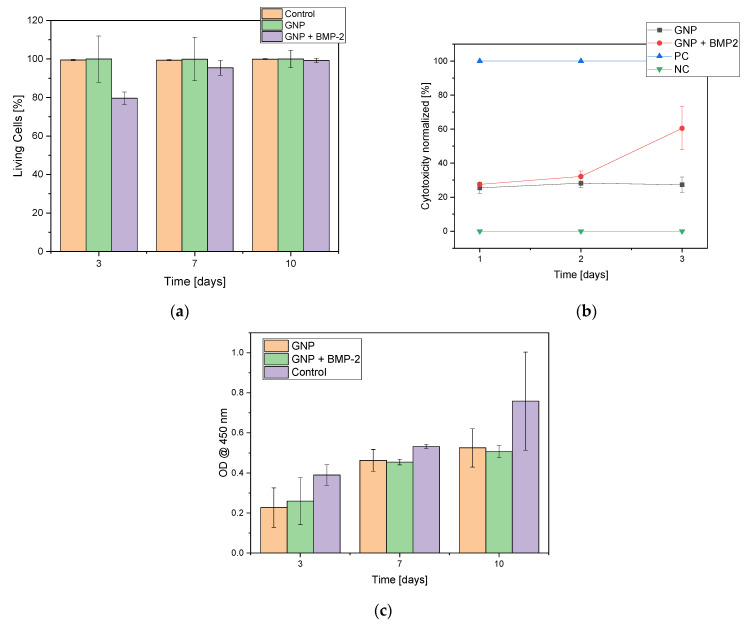
Overview of (**a**) live cells in the live/dead assay comparing “empty” GNPs, GNPs + BMP-2 with control; (**b**) cytotoxicity of negative control (NC = MG-63 cells), positive control (PC = Triton X), MG-63 cells with GNPs and MG-63 cells with GNP + BMP2; (**c**) Cell proliferation assay (WST-I). N = 3.

The cell count of MG-63 cells was found to increase continuously over the entire 7 day maintenance period (3, 7 and 10 days). Only isolated dead cells could be observed, especially within the first 24 h. Thereafter, only a living cell layer was observed. In Figure 4 the live dead staining of day 3 and day 7 is shown as an exemplar. The live/dead assay showed an increase in cell number over time. But in addition, the cell number with GNPs containing BMP2 was significantly lower compared to the “empty” non-loaded GNPs or control at all three time points (3, 7, 10 days). Nevertheless, a steady increase in the number of cells can be observed, both for the samples and the controls, while the number of dead cells remains at about the same level. The comparison of the living cells shows clear differences between the “empty” GNP, GNP + BMP2 and the controls. The control and the empty GNPs behaved in the same way, whereas the BMP2 loaded ones showed only 79.6 + 3.2% viable cells after 3 days instead of 99.5% for the controls and 99.9% for the empty GNPs (see Figure 5). From day 7 all three samples behaved the same way. Furthermore, agglomerates of GNPs could be detected by their strong fluorescence and subsequent ESEM investigations (see white arrow in Figure 4b and Figure S1).

In the early stages of the cell culture experiments we found that the cells preferred the GNPs with BMP-2 and agglomerated directly onto the GNPs instead of onto the Thermanox™ cover slips. These were then washed away during the media changes over the duration of the experiment. This also resulted in a lower number of living cells on the Thermanox™ cover slip in Figure 5a.

#### 2.3.2. Cell Proliferation Assay (WST-I)

In the WST-1 assay an increase in cell proliferation was observed over the duration of the experiment. In addition, it was observed that MG-63 cells prefered GNPs with BMP-2 over Thermanox™ cover slips and formed agglomerates with GNP + BMP-2 (see Supplement Figure S1).

#### 2.3.3. Lactate Dehydrogenase (LDH)

In the cytotoxicity assay, the constant cytotoxicity for the empty GNPs was shown to be 27.0 ± 0.1% compared to the cytotoxicity of GNPs with BMP-2, which increased after 2 days and reached a value of 60.5 ± 12.6% after 3 days (see Figure 5b).

### 2.4. Drug Release Experiments

For dual release, clindamycin was released from the ADA-GEL beads and the growth factor BMP-2 or its model substance FITC conjugated protein A from the GNPs within the beads.

#### 2.4.1. Clindamycin Release out of ADA-GEL Beads

**Figure 6:**
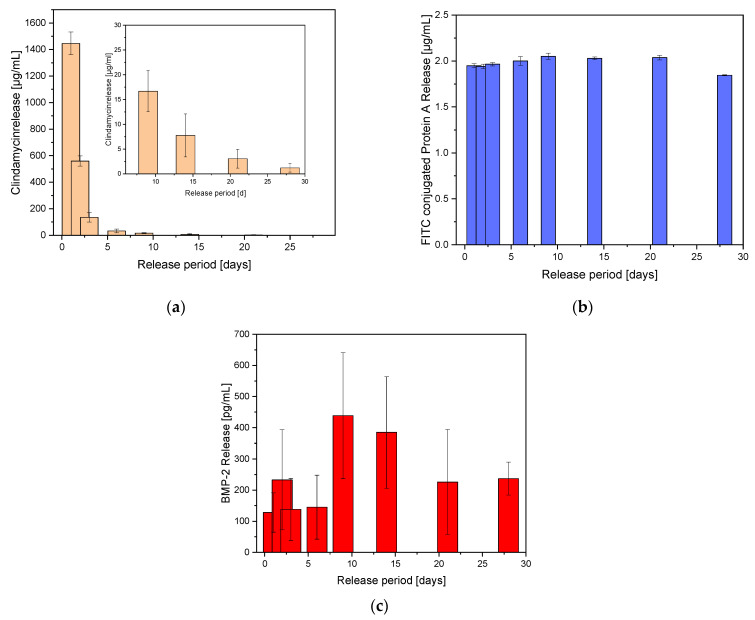
Dual release out of ADA-GEL beads containing GNPs.; (**a**) Clindamycin release over 28 days, the small figure represents an enlargement of the last release times. (**b**) FITC conjugated protein A released out of GNPs within the ADA-GEL beads. (**c**) BMP-2 release out of GNP within the ADA-GEL beads. N = 12.

Initially only the release of clindamycin will be considered. Figure 6a shows the release of clindamycin-HCl over a period of 28 days. On the last day, 3.1 ± 1.9 µg/mL of CLI has been released. This value was clearly above the MIC of 0.06 µg/mL for CLI against *Staph. aureus* [24]. In relation to the weighed amount, clindamycin was released with an initial burst release of 17% on average on the first day. After day 3, the amount released decreased sharply up to day 28 and was even below 1%. If the cumulative release curve was fitted according to Ritgers et al. [25] and the diffusion coefficient (n) was calculated, abnormal release rates were obtained for both the initial range (days 1–3) and the end of the release (days 6–28) (see supplement Figure S2a).

#### 2.4.2. FITC-Protein a Release out of GNPs within ADA-GEL Beads

In GNPs, the release of FITC conjugated protein-A was constant over the released period. A constant 1.98 ± 0.08 µg/mL was released at the measurement points. Looking at the cumulative release, a Fickian diffusion over the whole period (according to Ritger et al. [25]) with a diffusion coefficient of n = 0.5 was determined (see supplement Figure S2b). Figure 6b shows the FITC conjugated protein A release out of the GNPs within the ADA-GEL beads.

#### 2.4.3. BMP-2 Release out of GNPs within ADA-GEL Beads

Due to the use of GNPs, the expected burst release failed to appear. Instead, the release took place over the entire 28 days. Figure 6c shows the BMP-2 release out of the GNPs within the ADA-GEL beads. The cumulative release showed an almost linear course with similar daily release rates. According to Ritger et al. [25] the diffusion coefficient was 0.96, which corresponded to an anomalous release (see supplement Figure S2c).

## 3. Discussion

### 3.1. Characterization of GNP

According to Hathout and Metwally [26], the double de-solvation method was the best method to date for the production of GNPs. According to Coester et al. [14], GNPs produced by this method have a reduced tendency to agglomerate. Nevertheless, the reproducible production of GNPs was the greatest challenge of this work. Since gelatin was available as a heterogeneous mixture with different compositions, it was important to develop the individual production steps in such a way that they can always be carried out consistently. However, even if two approaches were carried out identically, the individual results often varied. The step that was least reproducible was the first de-solvation of the gelatin by the rapid pouring of acetone. This often resulted in chewing gum-like agglutinations of the gelatin, which, due to entrapped LMG, severely impaired the formation of GNPs. Therefore, this step was worked out by letting the acetone run in at the edge of the Erlenmeyer flask, since this was where the best results were achieved and the precipitation was also visually always uniform. Hamarat Sanlier et al. [13] described similar GNPs, but with a size of 300 nm. In their work they showed a similar distribution and arrangement of GNPs. Azizian et al. [23] and Modaresifar et al. [27] also described in their work the production and characterization of nanoparticles and their loading with growth factors such as BSA-bFGF, but on a chitosan basis. Their particles were 266 ± 5 nm in size. Similar to this work, they observed an increase in particle size due to loading. The size of the chitosan nanoparticles increased by 150 nm to 415 ± 9 nm. Feng et al. [28] and Zhai [22] investigated the influence of the pH value of the gelatin solution on the particle size of the GNPs. Feng et al. [28] achieved GNPs with a size of 400–450 nm with pH values of 8–11.

### 3.2. Inclusion Capacity

Azizian et al. [23] reported an inclusion capacity of the BSA or BSA-bFGF used in their work of 20 ± 1% and 21.3%. The inclusion capacity of the FITC conjugated protein A used in the present study was about twice as high at 46%. This was due to the fact that the FITC conjugated protein A of Invitrogen was already dissolved and stabilized with 10 mg/mL BSA. The BMP-2 was obtained as a lyophilized product from SinoBiological and the solvent was produced by our group without additional protein. The calculated inclusion capacity of the BMP-2 was 99.5%. Similar results were discussed by Poth et al. [29] in their work. They spoke of a complete inclusion of BMP-2 in their chitosan nanoparticles, but unfortunately did not specify an inclusion capacity.

### 3.3. Biocompatibility

Lee et al. [30] described GNPs in a similar order of magnitude as the ones we used (~150 nm). In the MTT test, they could not detect any significant toxicity based on the GNPs alone. However, they adressed a different application, i.e., the transport of siRNA in which their GNPs were taken up by the cells used. This differed from our study, in which our GNPs were detectable as agglomerates between the cells. Narayanan et al. [31] also described GNPs on a similar scale (70–220 nm) to those we used. They investigated the lymphocyte activation that occurred with any foreign body reaction. With the highest concentration of GNPs (1 mg/mL), no lymphocyte proliferation and therefore no foreign body reaction could be detected. The influence of BMP-2 on cells was sufficiently described in the work of Ribeiro et al. [32]. When looking at the results of cytotoxicity assay, a similar release pattern between the samples “empty” GNP and GNP + BMP2 was observed. Only on the last day of the study GNP + BMP-2 showed increased values compared to the empty GNPs. In a similar study [33], no negative effects have been observed. However, there only alginate and gelatin gels were used for the encapsulation of BMP-2. The values for cytotoxicity were quite high for GNPs with 27% and GNPs with BMP-2 at day 3 with a value of 60%. However, since there was a steady increase in cell numbers in the proliferation experiments and a significant increase in cell numbers in the live/dead, we assume that this is an effect related to the BMP-2 concentration released. It has been sufficiently described that high concentrations of BMP-2 tend to have a negative effect on cell growth [34]. We assume exactly such an effect within the first 72 h, but then BMP-2 levels subsequently returned to normal and led to an increase in cell numbers in the live/dead assay after 7 and 10 days. However, an interaction of GNPs with the cytotoxicity kit used cannot be completely excluded due to the high baseline values which were even observed for the unloaded GNPs. Some authors such as Li et al. [35] use only cell proliferation and live/dead analysis for the detection of cytotoxicity and avoid cytotoxicity investigations using specific kits. Abdelrady et al. [36] described the same method fabricating GNPs as in our work. They used GNPs in the same size range as our GNPs, 160 ± 9.78 nm, and also loaded GNPs with a drug during the 2nd desolvation stage and proved the safety and biocompatibility of empty GNPs.

Minardi et al. [37] also investigated the controlled release of BMP-2. But they used microspheres with a size of 23 ± 3 µm. As a proliferation test, they used a MTT assay where no significant difference between the control and the BMP-2 loaded microspheres could be detected. Similarly, Kim et al. [38] did not find any negative effects of the BMP-2-loaded PLGA nanoparticles on the surface of an HA scaffold when using human MSCs. Tseng et al. [39] used comparable sized GNPs between 180.6 ± 45.7 nm and 230.7 ± 84.6 nm in their work. The manufacturing process was very similar to ours, including the use of glutaraldehyde as a crosslinking agent. The Quick WST-1 test showed no significant difference between the different sizes of GNPs on cell proliferation of human corneal cells. But the proliferation was measured only at one time point, instead of three different time points compared to our work. Kuo et al. [40] also used GNPs in a similar size range (289.7 ± 6.8 nm to 360.0 ± 6.0 nm). They also used a WST-1 assay to determine cell proliferation. However, this test was only performed at two time points after 1 and 2 days only. In contrast to the MG-63 cells used by us, A-549 and H292 cell lines were used in their work.

### 3.4. Drug Release Experiments

The release experiments from the ADA-GEL beads showed similar results in terms of the release of CLI to those we have published in the past [41,42]. However, the use of the ADA-GEL gel resulted in an increase in burst release compared to our previously published work. By using alginate, the burst release could be reduced to 35% of the clindamycin amount weighed in. The fact that the ADA-GEL gel structure was different from that of the pure alginate changed the burst release and the total release. Nevertheless, the application of the drug release system, in another part of the project, showed very good antimicrobial efficacy [42]. Sarker et al. [43] also described in their work differences in the mechanical properties of the ADA-GEL gels compared to pure alginate gels. The release from the GNP beads showed a continuous release with a reduced burst release, as we intended. In comparison to other authors like Modaresifar et al. [27], where 75% of the loaded protein was released within the first 2 h, or Azizian et al. [23], where 80% of the release was completed after 4 h, we could show a continuous release over 4 weeks from the ADA-GEL beads with GNPs. However, we used bidest. water for our release experiments and Modaresifar et al. [27] and Azizian et al. [23] used PBS. In addition, the way we sampled (complete exchange of liquid) was different from sampling only a limited volume. Thus, we achieved higher values due to a higher diffusion pressure compared to authors who used only a part of the volume for the analysis.

However, the concentrations released were also very low in our work. In the case of BMP-2, very low concentrations are desirable due to the high potential for side effects [44]. Kim et al. [38] used BMP-2 loaded PLGA nanoparticles at the surface of a HA scaffold for their experiments. At 544 ± 39 nm the nanoparticles were significantly larger than those used by us. They were also able to demonstrate a release over 30 days in which 66% of the BMP-2 was released.

## 4. Conclusions

In the present work, GNPs were shown to be well suited for the release of BMP-2 or other large molecules such as FITC-conjugated protein A. A constant release (~2 µg/mL) corresponding to Fick’s diffusion was shown. In addition, dual release of clindamycin and BMP-2 from ADA-GEL beads was shown to be possible over an extended period of time (up to 4 weeks) with antimicrobial effective concentrations (CLI 25-fold above the MIC). In addition, the tissue compatibility of GNPs was demonstrated using various biocompatibility tests (L/D, WST-I, LDH) in cell culture with MG-63 cells.

## 5. Materials and Methods

### 5.1. Reagents and Materials

Gelatin type A (GEL), 175 Bloom, Alginic Acid for microbiological applications (Art.No. 71238), Ethylen glycol (Art.No. 324558) and Clindamycin-HCl (Art.No. PHR1159) (CLI) were obtained from Sigma-Aldrich (St. Louis, MO, USA). Acetone, HCl 0.1 M, Acetonitrile (HPLC grade) and Calcium chloride were obtained from Carl Roth (Karlsruhe, Germany). FITC conjugated protein A (molar ratio FITC/ protein A = 1.8; 2.5 mg/mL Protein A) was obtained by Invitrogen (Thermo Fisher Scientific, Waltham, MA, USA) and BMP-2 and the Human BMP-2 ELISA kit (KIT10426) from Sino Biological (Sino Biological, Inc., Beijing, China). Glutaraldehyde (GTA) was obtained from EMS (Electron Microscopy Sciences, Hatfield, PA, USA), gelatin type A 300 Bloom was kindly provided by GELITA (GELITA AG, Eberbach, Germany). Gelatin and Alginic acid were sterilized by means of a plasma sterilization process.

### 5.2. Manufacturing of the Gelatin Nanoparticles (GNPs)

The production of the GNPs was based on the protocol of Coester [14]. For the precipitation of the GEL, acetone was used according to Zhai [22] to produce GNPs with a diameter of 100 nm. For the production of GNPs, gelatin type A, 175 g Bloom was used. GEL was weighed into a 100 mL Erlenmeyer flask (Kern precision balance PCB250-2, Balingen, Germany) and a 5% aqueous GEL solution was mixed with aqua bidest. (0.625 g gelatin to 12.5 mL bidest. water). The GEL solution was homogenized on the heated magnetic stirrer (RCT basic, IKA^®^-Werke GmbH & CO. KG, Staufen, Germany) for 30 min, at 50 °C and 300 rpm. Parafilm was used to seal the Erlenmeyer flask. The first de-solvation was performed to separate the low molecular gelatin (LMG) from the high molecular gelatin (HMG). For this purpose, the temperature was switched off, the thermometer and magnetic stirring rod were removed and acetone (12.5 mL) was added to the gelatin solution evenly and rapidly at the slowest possible rate using a 25 mL disposable pipette, with a pipetboy (Integra Pipetboy 2, INTEGRA Biosciences AG, Zizers, Switzerland). It is important that the tip of the pipette touches the rim of the Erlenmeyer flask and that acetone flows into the solution via the inside of the glass flask. If acetone hits the GEL solution from above or if turbulence occurs in the vessel, flocculation and clumping of the GEL may occur. However, a uniform hydrogel-like, transparent precipitation of the HMG was desired, as the precipitated GEL lumps most probably also contained LMG, which could not form GNP. The Erlenmeyer flask was left standing for exactly 5 min, covered with parafilm. The supernatant was decanted and carefully rinsed with 1–2 mL bidest. water to remove any remaining LMG. Thermometer and magnetic stirring fish were replaced in the flask. HMG was solved in H_2_O. (12.5 mL) at 40 °C, 45 °C and 50 °C, 500 rpm for 30 min on the heated magnetic stirrer. With 0.1 M HCl the pH was adjusted to 2, 2.5 and 3 using a pH meter (Mettler Toledo Education line EL20, Schwerzenbach, Switzerland). The acetone was added drop by drop (40 mL) using a 100 mL dropping funnel. The connection between the dropping funnel and the Erlenmeyer flask was sealed with parafilm to prevent acetone from escaping. The opening of the funnel was also sealed and only shortly before the dripping process small openings were cut with a scalpel. If acetone escaped, no GNPs were produced or a large amount of acetone was needed for the particles to form. During the addition of acetone, the stirring speed was increased to 650 rpm. The formation of the nanoparticles can be recognized by the fact that the suspension has a bluish opalescent (Tyndall effect) and slightly turbid appearance. The nanoparticle suspension was stirred for another 10 min at 400 rpm, with the heater switched off, after the addition of acetone. To stabilize the resulting particles, 8% GTA (400 μL) was added. It is important here that the GTA is added drop by drop and over a period of at least 5 min, otherwise irreversible aggregation and cross-linking between the GNPs may occur. The nanoparticle suspension was stirred for 12 h, at room temperature and 350 rpm. The purification was performed by centrifugation (Avanti JXN-26, Beckman Coulter, Krefeld, Germany) with a fixed angle rotor (JLA-16.250, Beckman Coulter, Krefeld, Germany). Centrifuged at 15,000× *g*, 20 °C for 15 min, the supernatant was decanted and the pellet was resuspended in H_2_O by vortexing (Mini Vortex, Heathrow Scientific, Vernon Hills, IL, USA). The washing step was repeated twice, this time centrifuging at 16,000× *g*, 20 °C for 10 min. The purified GNP was dried with a lyophilisator (FreeZone 2.5 plus, Labconco, Kansas City, MO, USA), white powder was obtained when all of the water was sublimated.

#### Manufacturing of FITC-Protein A-Loaded GNPs and BMP-2 Loaded GNPs

The FITC-protein A GNPs were prepared as described, the addition of the FITC conjugated protein A was performed shortly before the second de-solvation step. It is important to note that FITC is light sensitive, therefore, the addition of the FITC-protein A was carried out in the absence of light. Concentrations of 1 and 2% *v*/*v* FITC-protein A in the GEL solution were prepared. For the final release experiments, the BMP-2-GNP (1 and 2% *v*/*v*) were prepared as described above, just like the FITC-protein A, the BMP-2 was added directly before the second de-solvation step. Therefore 0.4 mL the BMP-2 stock solution with 2.5 µg/mL was diluted with 0.6 mL bidest. water to reach a final concentration of 1 µg/mL. This solution was directly added before the second de-solvation step to the 125 mL solution (containing 0.625 g GEL).

### 5.3. Characterization of the GNP

#### 5.3.1. Characterization of GNP by ESEM

Size, structure and overall morphology of the GNP samples (empty, FITC-protein A and BMP-2 loaded), were examined using ESEM (FEI quanta 250 FEG, FEI, Hilsboro, OR, USA). The impact of variations in the production method (temperature, pH, stirring speeds and times of addition of the crosslinker) on the formation of GNPs, size, shape and aggregation were investigated. For this purpose, double-sided adhesive polycarbonate-based conductive tabs were glued to pin sample plates made of aluminium. The freeze-dried samples in powder form could thus be fixed on the adhesive surface and were sputtered with gold before ESEM measurement (JFC-1200 fine coater, Jeol, Freising, Germany) to increase the conductivity and stability of the samples in high vacuum.

#### 5.3.2. Determination of the Inclusion Capacity

To assess how much protein was actually present in the nanoparticles, the supernatant obtained after centrifugation was measured with the fluorimeter (Ensight Multimode Plate Reader, PerkinElmer, Rodgau, Germany). The Kaleido software was used to obtain the data for quantitative analysis. A calibration curve was generated based on levels within the supernatant, which could then be used to approximate protein content within the solvent. Acetone and water in a ratio of 4:1 (*v*/*v*), plus a quarter of the amount of GTA were used. The greatest uncertainty was found for the amount of GTA, since the amount of unreacted GTA after the crosslinking process was unknown. The difference between the amount of FITC-protein A used and the amount in the supernatant was defined as the amount included in the GNPs. In order to quantify how much BMP-2 was present in the produced GNPs, the supernatant obtained after the first centrifugation was analyzed using an ELISA. The evaluation of the color reaction was performed with the UV/Vis spectrometer (Spectrostar Nano, BMG Labtech, Ortenberg, Germany). A calibration curve was generated using the BMP-2 standard of the kit. The difference between the amount of BMP-2 used and the amount in the supernatant was defined as included BMP-2 in the GNPs.

(1) $$\mathrm{IC}=\frac{m({\mathrm{FITC}}_{\mathrm{total}})-m({\mathrm{FITC}}_{\mathrm{supernatant}})}{m({\mathrm{FITC}}_{\mathrm{total}})}$$

IC: Inclusion capacity;

m(FITC_supernatant_): Mass of FITC-protein A at supernatant;

m(FITC_total_): Total mass of FITC-protein A.

### 5.4. Biocompatibility

In order to show that the GNPs were biocompatible with living cells and that after purification no cytotoxic residues such as acetone or GTA from the production steps were present, a cell culture experiment was carried out. MG-63 cells (ATCC CRL 1427) were used. For each sample 50,000 cells were placed in a 12-well plate on glass plates, which were previously coated with polylysine. The cells were cultivated overnight with Dulbecco’s Modified Eagle Medium (DMEM F12 containing F12 nutrient and the additions of 1% penicillin/streptomycin (P/S, Sigma Aldrich (now Merck), Darmstadt, Germany) and 10% fetal bovine serum (FBS, Merck, Darmstadt, Germany) in a New Brunswick Galaxy 170 R incubator (Eppendorf, Hamburg, Germany) at 37 °C and a CO_2_ saturation of 5%. The next day, a production batch (from originally 1.25 g gelatin) GNP were washed in 1 mL ethanol (60%) to prevent cell culture contamination. The GNP were dissolved in the culture medium and pipetted to the cells. Nunc™ Thermanox™ Coverslip (Thermo Fisher Scientific, Waltham, MA, USA) membranes were used as a control. For each time point at least three samples were used and all experiments were repeated at least three times.

#### 5.4.1. Live/Dead Assay

A total of 3 samples for each day and one blank sample (without GNP) for day 3, 7 and 10 were used for the live/dead assay. For the live/dead assay (L/D), the cells were stained with Calcein-AM and EthD-III (out of Live/Dead Cell Staining Kit II (PromoCell, Heidelberg, Germany)) on the respective days and observed under the fluorescence microscope (Olympus BX 51, Olympus, Hamburg, Germany), the evaluation was performed with the Stream Motion Software Version 1.7.1 from Olympus. Living cells exhibited a green fluorescence under blue light, and dead cells a red fluorescence. In a further experiment the influence of the released BMP-2 on the MG-63 cells was investigated. The L/D assay was repeated with GNPs loaded with BMP-2. This was followed by incubation in the incubator for the various experiments.

#### 5.4.2. Cell Proliferation Assay

Thermanox™ Coverslip membranes were used as a control. All samples and controls were equally covered with 50,000 cells in 200 µL. The cells were incubated for 4 h at 37 °C with a CO_2_ saturation of 5% in the incubator for 3, 7 and 10 days. At the end of this period, 400 µL of the DMEM-F12 complete medium were added to each sample and incubated. After 24 h at day 0, 100 µL resuspended GNP + BMP2 were added. A medium change with the DMEM-F12 with the 10% FBS and 1% P/S additives was performed for days 7 and 10. For each WST evaluation the medium was aspirated, and the wells were washed three times with PBS. The samples and the Thermanox™ Coverslips were then transferred to a new well, and then 400 µL of the DMEM-F12 phenol red free (Art. No. 11039-021, Gibco, Grand Island, NE, USA) with the 1% P/S and 1% fetal bovine serum (FBS, Merck, Darmstadt, Germany) additives were added to the wells with the sample. 400 µL of the medium were added to the previously used empty sample wells, Positive Control (C + R), Empty Control Well (C+), and Blank. The blank contained only the DMEM medium without phenol red and was measured to account for background absorption. 10% WST reagent (Art. No. 05015944001, Roche, Basel, Switzerland) was added to the corresponding volume of medium. Thus, 250 µL WST were added to the sample wells, and 40 µL were added to the old wells, the blank wells, and the positive control (C + R). This was incubated in an incubator at 37 °C for 2 h. After this time, the liquids were transferred into a 96 well plate. 100 µL of each solution were added to three separate wells. The absorption was then measured at 450 nm using a Spectrostar Nano microplate reader (BMG Labtech, Ortenberg, Germany). In a further experiment the influence of the released BMP-2 on the MG-63 cells was investigated. The WST-I was repeated with GNPs loaded with BMP-2.

#### 5.4.3. Lactate Dehydrogenase (LDH) Assay

Each experiment assessed three Thermanox™ cover slips, a negative control (cells only), a positive control (Triton X), and a blank to account for background absorbance in the ELISA reader. The experiments were repeated at least three times. A 200 µL cell solution containing 50,000 cells was seeded onto each scaffold, and a 100 µL cell solution containing 50,000 cells was seeded onto the Thermanox™ cover slips. One well was left empty for use as a blank. The well plate was placed in an incubator at 37 °C with 5% CO_2_ for 4 h. Following incubation, 400 µL of DMEM-F12 phenol red free with the 1% P/S and 1% FBS additives were added into the sample-wells and control-wells. Since FBS itself contains LDH, a concentration of 10% in the medium might have triggered background absorption. Therefore, only a concentration of 1% FBS was added to the medium. For the positive controls, 1% Triton × 100 (Art. No. X100, Sigma Aldrich, Saint Louis, MO, USA) was added to the DMEM-F12 medium with 1% P/S and 1% FBS to kill the cells. The LDH experiments were carried out at 24, 48 and 72 h following seeding and the same procedure was repeated at each interval: Three 100 µL samples were taken from each well into a 96 well plate. An LDH reagent (100 µL) was added to each well in use, and the plate was incubated in darkness at room temperature for 30 min. Following incubation, the plate was placed in a Spectrostar Nano microplate reader (BMG Labtech, Ortenberg, Germany), and absorbance was measured at a λ of 490 nm with a reference λ of 600 nm. In a further experiment the influence of the released BMP-2 on the MG-63 cells was investigated. The LDH was repeated with GNPs loaded with BMP-2.

### 5.5. ADA-GEL Hydrogel

#### 5.5.1. Preparation of Alginate-di-Aldehyde (ADA)

ADA was produced according to the method developed by Sarker et al. [45]. For this purpose, sodium alginate was dissolved in ethanol (99.8%) and sodium periodate dissolved in aqua bidest. was added. For the oxidation reaction, stirring was carried out for 6 h under exclusion of light (beaker was wrapped with aluminum foil). The reaction was stopped with ethylene glycol and stirring was continued for 30 min. The ADA was dialysed for 7 days against aqua bidest. to remove any remaining sodium periodate by using the dialysis system Spectra/Por (Repligen, Boston, MA, USA) with standard RC dialysis membranes (6–8 kD MWCO). The bidest. water was changed twice a day. After dialysis, the ADA was dried in a lyophilisator (FreeZone 2.5, Labconco, Kansas City, MO, USA) for another seven days.

#### 5.5.2. Crosslinking ADA and GEL

The cross-linking of the aldehyde groups produced by oxidation with the amino groups of the gelatin always took place shortly before the gel was used and is described in the following experiments.

### 5.6. ADA-GEL Beads with Drugs

#### 5.6.1. ADA-GEL Beads with Clindamycin and FITC-Protein A-Containing GNPs

A 5% *w*/*v* ADA solution was prepared by homogenizing freeze-dried ADA with aqua bidest. water for several hours on a magnetic stirrer in a beaker. Meanwhile, a 5% GEL solution was prepared from GEL A, 300 g Bloom (GELITA AG, Eberbach, Germany) with aqua bidest. water at 37 °C on the magnetic stirrer. Clindamycin was added to the gelatin after homogenization to achieve a final concentration in the beads of 50 mg/mL. Since clindamycin is light sensitive, the beaker was wrapped in aluminum foil. The amount of FITC-protein A GNPs from 2 production batches of 1.25 g each of initial GEL mass was added. GNP prepared with 2% FITC-protein A was used. The GNPs were added to the homogenized ADA. The clindamycin-containing gelatin and the ADA containing GNP were mixed together (1:1) and carefully dripped with the beaker into a 30 mM calcium chloride solution [19]. In addition to the active substance containing beads, hydrogel beads were prepared as blank samples without drugs.

#### 5.6.2. ADA-GEL Beads with CLI and BMP-2 Containing GNP

The beads were prepared with CLI and BMP-2 containing GNPs as described in 2.6.1. Before adding the gelatin, GNPs with BMP-2 from a batch of 1.25 g of gelatin (twice as much as described in 2.2) was added to the ADA. After the addition of the gelatin containing CLI, the mixture was homogenized under light for 30 s. The beads were then prepared by dropping the ADA-GEL into 30 mM CaCl_2_ solution.

### 5.7. Drug Release Experiments

All drug release experiments were performed with a minimum of 10 samples and repeated at least three times.

#### 5.7.1. Drug Release from ADA-GEL Beads

1 g of the CLI loaded beads were stored, after being weighed with a precision balance (Secura 125-1CEU, Sartorius, Göttingen, Germany), with 3 mL bidest. water in 5 mL Eppendorf Tubes, at 37 °C for 28 days in an oven (Memmert IN 75, Schwabach, Germany). After 1, 2, 3, 6, 9, 14, 21 and 28 days, samples were taken and the liquid was completely removed and replaced by aqua bidest. The obtained liquid was frozen at −20 °C until analysis by HPLC.

#### 5.7.2. Dual Drug Release from ADA-GEL Beads

The dual release (CLI out of the ADA-GEL beads, BMP-2 out of GNPs within the ADA-GEL beads) was carried out here according to the same principle as described above. 1 g of loaded ADA-GEL beads was placed in a 5 mL Eppendorf tube with 3 mL bidest. water. The release was tested at 37 °C for 28 days. After 1, 2, 3, 6, 9, 14, 21 and 28 days, samples were taken, the complete liquid was removed once again and replaced by bidest. water. The samples were frozen at −20 °C in the same way as the release tests from the beads until they were analyzed by HPLC or fluorimeter (FITC-prot A) and ELISA kit (BMP-2).

#### 5.7.3. Quantitative Analysis

The released clindamycin from the ADA gelatin beads was quantitatively determined by HPLC. The HPLC System (Shimadzu, Kyoto, Japan) consisting of 2 Nexera XR LC-20AD pumps and a SIC-30AC autosampler, CTO 20 AC column oven, DGU-20A5R Degasser, SPD-M20A PDA detector, RF 20A fluorescence detector and a CBM-20A controller. A reversed-phase column of butyl-modified silica gel was used as a separation column (NUCLEOSIL 300-5 C4, 5 μm, 250 × 4.6 mm, Macherey-Nagel, REF 761989.30, Düren, Germany). An acetonitrile/sodium hydrogen phosphate solvent with pH of 3.5 at a the ratio 29:71 was used according to Batzias et al. [46]. Flow rate was 0.66 mL/min at 25 °C. Clindamycin was eluted after 3.5 min and registered by a PDA detector at 193 nm. The released FITC-protein A concentration was measured with a fluorimeter (Perkin Elmer EnSight, Waltham, MA, USA) at 490 nm excitation and 525 nm emission. The determination of the BMP-2 concentration was performed with an ELISA kit from Sino Biological according to their protocol. The 96 well plate already coated with a BMP-2 antibody by the manufacturer was first washed 3 times with 200 µL wash buffer. 100 µL of the samples were then pipetted into the wells and incubated at room temperature for two hours. The washing step was repeated 3 times before 100 µL of the detection antibody against BMP-2 were added conjugated with horseradish peroxidase. This was incubated for one hour at room temperature. Washing was repeated 3 times before the dye solution was added. After 20 min 50 µL of the Stop Solution were added to stop the color reaction. Using a UV/Vis spectrometer (SpectroStar Nano, BMG Labtech, Ortenberg, Germany), the color reaction was quantitatively evaluated using a calibration curve (2500…31.5 pg/mL) at λ = 450 nm.

#### 5.7.4. Kinetics Model

The release kinetics of CLI, FITC, and BMP-2 were fitted to a cumulative diagram according to the following Ritger [47] exponential relationship:

(2) $$\frac{M_{t}}{M_{\infty }}={\mathrm{kt}}^{n}$$

where M_t_/M_∞_ = fractional solute release; t = release time; k = a constant; and n = diffusional exponent characteristic of the release mechanism. In the case of pure Fickian release, the exponent n has limited values 0.50, 0.45 and 0.43 for release from slabs, cylinders and spheres.

### 5.8. Statistical Analysis

Data was expressed as mean values ± standard deviation of the mean and analyzed by one-way analysis of variance (ANOVA). The level of statistical significance was set at *p* < 0.05. For statistical calculations, Origin 2020 Professional SR1 (OriginLab, Northampton, MA, USA) was used.

## Data Availability

The data presented in this study are available on request from the corresponding author.

## Supplementary Materials

The following supporting information can be downloaded at: https://www.mdpi.com/article/10.3390/gels8060365/s1. Figure S1: Image (live/dead staining) of agglomerated GNP-BMP2 with MG-63 cells; Figure S2: Cumulative releases, fit according to Ritger et al. [25]; (a) Clindamycin; (b) Conjugated FITC-protein A, with a diffusion coefficient n = 0.5 for Fickian’ diffusion; (c) BMP-2 in relation to the BMP-2 amount used. According to previous published work [41], the fitting for the CLI release was split into a beginning part (n_B_) and final part (n_F_), both with anomalous diffusion (n_B_ = 0.35 and n_F_ = 0.01).
