# Supplementary material for: Gelatin Nanoparticles for Targeted Dual Drug Release out of Alginate-di-Aldehyde-Gelatin Gels
Source: Gels. 2022 Jun 8;8(6):365. doi: 10.3390/gels8060365 (PMC9222291; doi:10.3390/gels8060365)
Supplement: Supplementary file 1 [file gels-08-00365-s001.zip › gels-1742578-supplementary.pdf]

gels

MDPI

Article

Gelatin Nanoparticles for Targeted Dual Drug Release out of Alginate-di-Aldehyde-Gelatin Gels

Sophie Schrade [1,2], Lucas Ritschl [1], Regine Süss [2], Pia Schilling [1] and Michael Seidenstuecker [1,*]

[1] G.E.R.N. Center of Tissue Replacement, Regeneration & Neogenesis, Department of Orthopedics and Trauma Surgery, Medical Center - Albert-Ludwigs-University of Freiburg, Faculty of Medicine, Albert-Ludwigs-University of Freiburg, Hugstetter Straße 55, 79106 Freiburg, Germany; sophie.schrade@uniklinik-freiburg.de (S.S.); lucas.ritschl@uniklinik-freiburg.de (L.R.); pia.schilling@uniklinik-freiburg.de (P.S.)
[2] Institute of Pharmaceutical Sciences, Albert-Ludwigs-University of Freiburg, Sonnenstr. 5, 79104 Freiburg, Germany; regine.suess@pharmazie.uni-freiburg.de
* Correspondence: michael.seidenstuecker@uniklinik-freiburg.de

Supplementary Materials

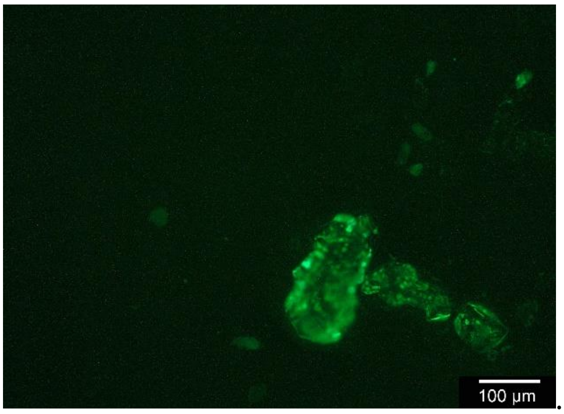

Figure S1. Image (Live Dead staining) of agglomerated GNP-BMP2 with MG-63 cells.

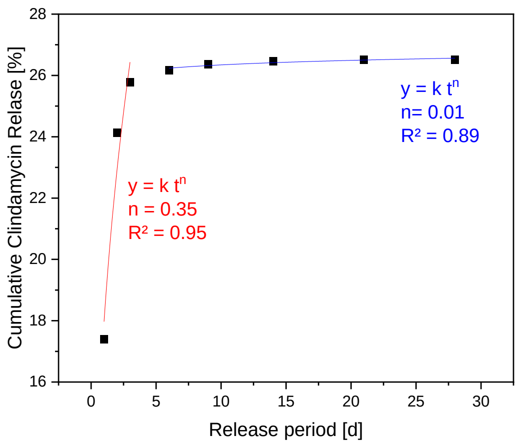

(a)

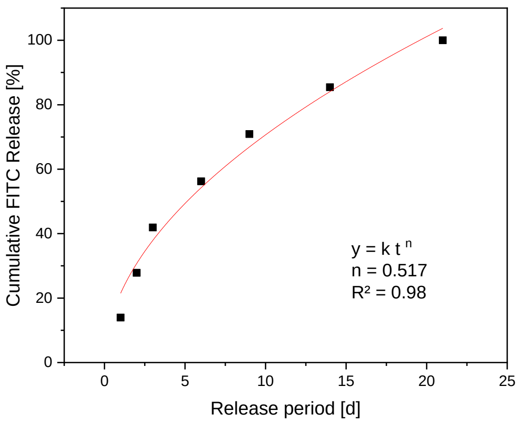

(b)

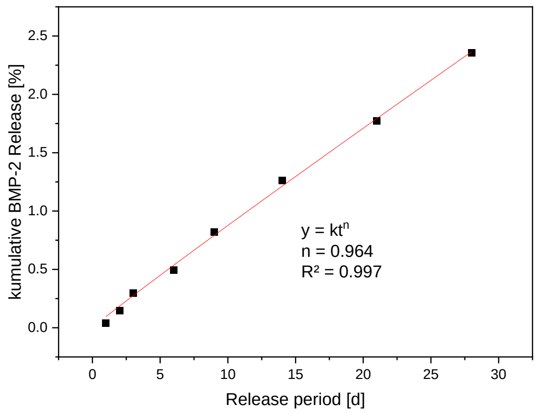

(**c**)

Figure S2. Cumulative releases, fit according to Ritger et al. [28]; (**a**) Clindamycin; (**b**) Conjugated FITC-protein A, with a diffusion coefficient n=0.5 for Fickian' diffusion; (**c**) BMP-2 in relation to the BMP-2 amount used. According to previous published work [44], the fitting for the CLI release was split into a beginning part (n_B) and final part (n_F), both with a normal diffusion (n_B = 0.35 and n_F = 0.01).
